# Sensitivity Differences and Biochemical Characteristics of *Laodelphax striatellus* (Fallén) to Seven Insecticides in Different Areas of Shandong, China

**DOI:** 10.3390/insects13090780

**Published:** 2022-08-29

**Authors:** Yannan Xue, Chang Liu, Dongmei Liu, Wenjuan Ding, Zhaoge Li, Junli Cao, Xiaoming Xia

**Affiliations:** 1 College of Plant Protection, Shandong Agricultural University, Taian 271018, China; 2 Shanghai Yuelian Chemical Industry Co., Ltd., Shanghai 201416, China

**Keywords:** *Laodelphax striatellus* Fallén, nicotinic acetylcholine receptor, sensitivity, biochemical characteristics

## Abstract

**Simple Summary:**

The sensitivity detection of pests to insecticides is useful to the strategies of integrated pest management (IPM) in the field. The sensitivities of six *Laodelphax striatellus* Fallén populations from different areas in Shandong, China to seven insecticides acting on the nicotinic acetylcholine receptor (nAChR) were investigated. The biochemical characteristics of different populations were also studied. The results showed that all the field populations are sensitive to clothianidin, nitenpyram, and triflumezopyrim, but some field populations have developed resistance to other insecticides. The populations showed different metabolic enzyme contents caused by the expression of related genes, and no known mutations in the target gene nAChR β1 subunit were found in any of the populations. These results provide valuable information for the management strategies of *L. striatellus* in field.

**Abstract:**

*Laodelphax striatellus* Fallén is one of the main pests that can severely harm rice, corn, and wheat. Insecticides acting on the nicotinic acetylcholine receptor (nAChR) are the main type of pesticides used for the control of *L. striatellus* in Shandong Province, a major grain-producing region in China. In this study, the rice seedling dipping method was used to determine the sensitivities of six field *L. striatellus* populations in Shandong to seven insecticides acting on nAChR. The results showed that all the field populations were sensitive to clothianidin, nitenpyram, and triflumezopyrim, and the Jiaxiang population exhibited the lowest resistance ratio (RR) to imidacloprid, dinotefuran, sulfoxaflor, and thiamethoxam. The Donggang population showed a medium-level resistance to imidacloprid, with the highest RR of 17.48-fold. The Yutai population showed low-level resistance to imidacloprid and thiamethoxam, with RRs of 7.23- and 7.02-fold, respectively. The contents of cytochrome P450 monooxygenase (P450s), carboxylesterase (CarE), and glutathione S-transferase (GST) were the highest in the Donggang population and the lowest in the Jiaxiang population. The P450 gene *CYP314A1* and the CarE gene *LsCarE12* were highly up-regulated in all populations. No mutations of V62I, R81T, and K265E in the nAChR β1 subunit were found in any of the populations. These results provide valuable information for the strategies of resistance management of *L. striatellus* in the field.

## 1. Introduction

*Laodelphax striatellus* Fallén (Hemiptera: Delphacidae) is an important pest of rice, corn, and wheat [1]. It is mainly distributed in Asia and North Africa, and in warm temperate areas of Europe [2]. Both the adults and nymphs of *L. striatellus* can suck plant sap from the phloem, consuming plant nutrients [3]. In addition, *L. striatellus* can transmit rice stripe virus and maize rough dwarf virus, which damage crops and cause severe yield losses [4].

Neonicotinoids, sulfonimines, and mesoionic insecticides all act on the nicotinic acetylcholine receptor (nAChR), and are widely used in the control of *L. striatellus* [5]. However, due to the widespread and frequent use of insecticides, *L. striatellus* has developed resistance to these insecticides, including imidacloprid, nitenpyram, and thiamethoxam, leading to control difficulties [6,7,8]. In Shandong Province, the main maize-producing area in China, the sensitivity of *L. striatellus* in the field to insecticides acting on nAChR is not clear.

Studies have shown that the reduction of *L. striatellus* sensitivity to insecticides is mainly due to the enhanced activity of metabolic enzymes, including cytochrome P450 monooxygenase (P450s), carboxylesterase (CarE), and glutathione S-transferase (GST) [9,10]. This enhancement of enzyme activity is generally caused by the overexpression of related genes [11].

The overexpression of *CYP425B1* and *CYPDE1* leads to increased P450s activity, which is responsible for imidacloprid resistance in *L. striatellus* [12]. A previous study has reported that up-regulation of *LsCarE1* and *LsCarE2* mediates the resistance of *L. striatellus* to chlorpyrifos [13]. GST can prevent and repair oxidative damage caused by exogenous substances in *Drosophila* [14]. The *NlGSTs1*, *NlGSTs2*, *NlGSTe1*, and *NlGSTm1* have been shown to play important roles in the resistance of *Nilaparvata lugens* Stål to imidacloprid [15].

Target resistance caused by the mutation of target genes is another important mechanism of insecticide resistance in pests [16]. Mutations sites at V62I, R81T, and K264E located in the nAChR β1 subunit are associated with resistance to imidacloprid and acetamiprid in insects [17,18,19].

In this study, the sensitivity of six *L. striatellus* populations, collected from different areas in Shandong, to seven insecticides (imidacloprid, nitenpyram, thiamethoxam, clothianidin, dinotefuran, sulfoxaflor, and triflumezopyrim) acting on the nAChR was detected. The contents of P450s, CarE, and GST, the related metabolic gene expression and target gene mutations were also determined. The results could be useful in the application of insecticides for the management of *L. striatellus* in the field.

## 2. Materials and Methods

### 2.1. Insects

*L. striatellus* populations were collected from six different locations: Donggang (119.35° E, 35.28° N), Tancheng (118.28° E, 34.52° N), Yutai (116.56° E, 34.97° N), Jiaxiang (116.304° E, 35.41° N), Daiyue (117.03° E, 35.97° N), and Jiyang (117.23° E, 37.04° N) in Shandong Province, China, during September to October 2020. The relatively susceptible strain (SS) was maintained in laboratory for more than five years without exposure to any chemicals [1]. All insects were fed with ‘Wuyujing 3’ rice seedlings under laboratory conditions of 25 ± 1 °C, 70–80% relative humidity, and a 16:8 h L:D photoperiod [20].

### 2.2. Chemicals

Imidacloprid (96%) was provided by Shandong Weifang Rainbow Chemical Co., Ltd. (Weifang, China). Nitenpyram (98%), thiamethoxam (98%), clothianidin (98%), and dinotefuran (99.1%) were supplied by Shandong United Pesticide Industry Co., Ltd. (Jinan, China). Triflumezopyrim (96%) was supplied by DuPont Company (Shanghai, China). Sulfoxaflor (95.9%) was supplied by Dow Agroforestry Corporation (Beijing, China).

### 2.3. Bioassays

The toxicities of the insecticides to *L. striatellus* were determined using the rice seedling dipping method [20]. The insecticides were dissolved in acetone and diluted with deionized water containing 0.1% Triton to a serial concentration. The serial concentrations of each tested pesticide are shown in Supplementary Materials S1. Control groups were treated with deionized water containing 0.1% Triton. Five rice seedlings were immersed in the solution for 30 s, and then removed and dried naturally in the shade. The roots of the rice seedlings were wrapped with wet absorbent cotton and placed into a 20 mm × 200 mm glass test tube. Thirty third-instar nymphs were transferred into each tube and then sealed with four layers of 20-mesh gauze. Ninety insects were treated for each concentration performed with three replicates. Finally, all treatments were placed in an incubator following the above feeding conditions, and the mortality was checked at 72 h [21].

### 2.4. Enzymes Contents Assays

The P450s, CarE, and GST contents were detected according to the kit’s instructions (Hengyuan Biotechnology Co., Ltd., Shanghai, China), using a double-antibody sandwich method based on immunoassay principles [22,23,24]. The kit numbers are HB905X-QT, HB863X-QT, and HB593X-QT, respectively. Third-instar nymphs per population were used for the preparation of the enzyme solution. Each population includes three replications (30 insects per replicate). The protein content of the enzyme solution was determined using the BCA protein kit (Beyotime Biotechnology Co., Ltd., Shanghai, China) [25].

The P450s, CarE, and GST contents were calculated according to the standard curve and protein content, and the results are expressed as μg/g prot. The specific determination steps of P450s, CarE, and GST contents are in Supplementary Materials S2.

### 2.5. Detection of Gene Expression Levels

According to the previous transcriptome sequencing results (no published), the expression of seven P450 genes, eight CarE genes, and eight GST genes in all seven populations was determined by the real-time quantitative PCR (RT-qPCR) [11], and the SS was used as a control group. The chemical reagents used in this section were purchased from Novizan Biotechnology Co., Ltd. (Nanjing, China).

Twenty-five third-instar nymphs were collected from each population to extract total RNA, and each population contained three biological repetitions. The total RNA was extracted using RNA-esay^TM^ Isolation Reagent (Novizan Biotechnology Co., Ltd., Nanjing, China), and 1 μg of total RNA was reverse transcribed to cDNA synthesis by using reverse transcription kit (Novizan Biotechnology Co., Ltd., Nanjing, China).

The ChamQ^TM^ Universal SYBR^®^ qPCR Master Mix was used to carry out the qRT-PCR reaction in a QuantStudio^TM^ 3 system (Thermo ABI, Santa Clara, CA, USA). The reference gene (GAPDH) and primers are shown in Table 1. Relative quantifications were achieved based on using the 2^−ΔΔCT^method [26].

### 2.6. Detection of Gene Mutation Sites in nAChR β1 Subunit

Gene sites V62I, R81T, and K264E of the nAChR β1 subunit were selected for mutation detection based on the previously reported gene mutation of insecticide resistance [18,19]. Based on the complete mRNA sequence (GenBank: MF612140.1, NCBI) of nAChR β1 subunit of *L. striatellus*, the primer sequences were designed to amplify a length range of 31 bp–1381 bp fragments containing the above three mutation sites (Table 1).

A third-instar nymph was taken, and an ultra-trace total RNA extraction kit (Nobel Lai Biotechnology Co., Ltd., Beijing, China) was used to extract total RNA. Then, 1 μg of total RNA was taken for cDNA synthesis. Finally, 2×Phanta Flash Master Mix (Dye Plus) (Novizan Biotechnology Co., Ltd., Nanjing, China) was used to amplify the target gene in the PCR system (Thermo Fisher Scientific, Santa Clara, CA, USA). The PCR product was sent to a company (Sangon Biotechnology Co., Ltd., Shanghai, China) for sequencing.

### 2.7. Statistical Analysis

All the results of the sensitivity detection of *L. striatellus* to the pesticide were processed by the SPSS software (V. 20.0 for Windows, SPSS Inc., Chicago, IL, USA). The insecticide treatment mortality was corrected with control mortality using Abbott’s formula. The median lethal concentration (LC_50_), slope, 95% confidence interval (CI), *χ*^2^, and coefficient of determination (R^2^) were calculated using probit analysis and the chi-square test. R^2^ ranges from 0 to 1, with 1 indicating the highest goodness of fit of the toxicity data to the linear regression equation. The differences in metabolic enzyme activities and gene expression levels were analyzed by one-way ANOVA with Tukey’s test method at 0.05 levels. The target gene mutation sites were analyzed using DNAman software (V. 9.0, Lynnon Biosoft Inc., San Ramon, CA, USA).

The LC_50_ value of the susceptibility baseline of each insecticide to *L. striatellus* is shown in Table 2. The resistance ratio (RR) (LC_50_ of field populations/LC_50_ of SS or baseline) was calculated and used to clarify the resistance level: low (RR = 5–10-fold), medium (RR = 10–100-fold), and high (>100-fold) [27].

## 3. Results

### 3.1. Insecticide Sensitivity in Different Populations of L. striatellus

The results showed that the Donggang and Yutai populations had medium- and low-level resistance to imidacloprid, with RRs of 17.48- and 7.23-fold, respectively. However, no imidacloprid resistance was determined in the other four populations (Table 3). The Donggang, Yutai, and Jiyang populations showed low-level resistance to dinotefuran, with RRs of 5.84-, 7.81-, and 5.04-fold, respectively (Table 4). A low level of sulfoxaflor resistance was also detected in the Tancheng and Jiyang populations, with RRs of 5.20- and 5.35-fold, respectively (Table 5). However, only the Yutai population had low-level resistance to thiamethoxam, with an RR of 7.02-fold (Table 6). However, all six field populations were sensitive to nitenpyram, clothianidin, and triflumezopyrim (Table 7, Table 8 and Table 9).

### 3.2. Metabolic Enzymes Contents in Different Populations of L. striatellus

**Figure 1:**
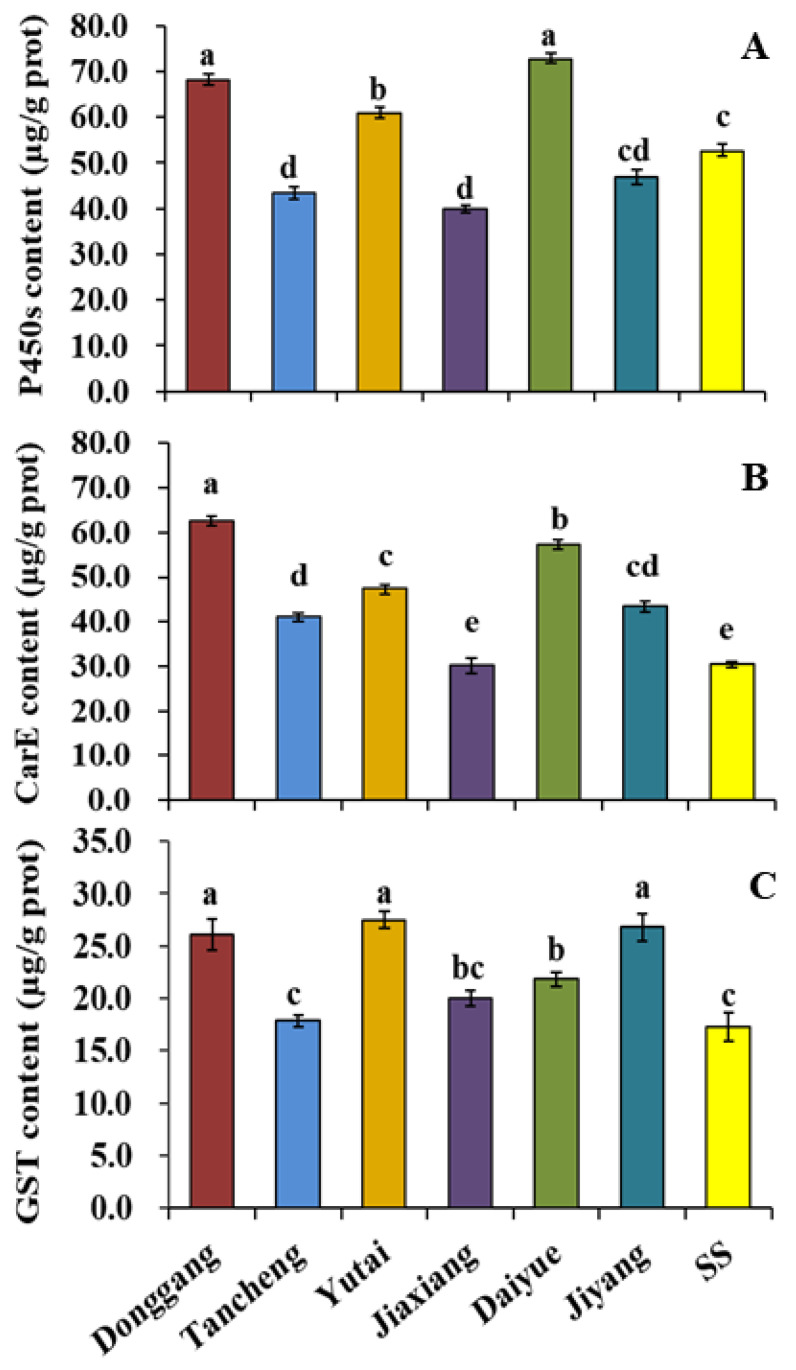
P450s (**A**), CarE (**B**), and GST (**C**) contents of six *L. striatellus* populations in Shandong Province. The data are the mean ± SE (*n* = 3), and different letters on the bars indicate significant differences (*p* < 0.05).

The contents of P450s were the highest in the Donggang and Daiyue populations, followed by the Yutai population, and the lowest in the Tancheng and Jiaxiang populations, compared with the SS (Figure 1A). P450s contents in the Jiyang population showed no significant differences with SS.

The Donggang population showed the highest CarE contents in all the tested populations, followed by the Daiyue and Yutai populations (Figure 1B). The Jiaxiang population exhibited the lowest CarE contents, and there was no significant difference with the SS. The contents of CarE in the Tancheng and Jiyang populations were significantly higher than in the SS.

The GST contents results (Figure 1C) showed that the Donggang, Yutai, and Jiyang populations had the highest contents. The Daiyue population had high GST content. The Tancheng and Jiaxiang populations exhibited the lowest GST contents, and showed no significant differences with SS.

### 3.3. Gene Expression Levels in Different Populations of L. striatellus

**Figure 2:**
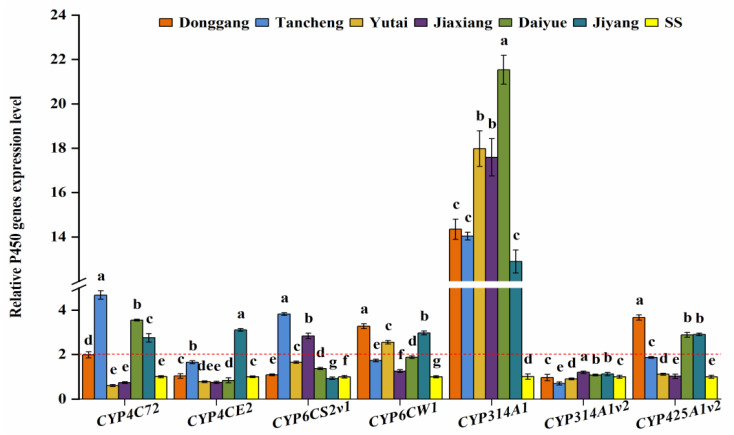
The relative expression levels of seven P450 genes in six *L. striatellus* populations from Shandong Province compared with SS (red dotted line means 2-fold overexpression level). The data are the mean ± SE (*n* = 3), and different letters on the bars indicate significant differences (*p* < 0.05).

In order to explore the potential metabolic enzyme genes that might be associated with insecticide sensitivity, the relative expression levels were determined for seven P450 genes, eight CarE genes, and eight GST genes in different populations of *L. striatellus*. The P450 gene results showed that the relative expression levels of different P450 genes were significantly different in different populations (Figure 2). Compared with SS, the relative expression levels of *CYP314A1* were significantly up-regulated more than 10-fold in all the field populations, with 12.89–21.53-fold, however, *CYP314A1v2* was less than 2-fold in all the field populations. *CYP4C72* was significantly up-regulated in the Tancheng, Daiyue, and Jiyang populations with 4.68-, 3.55-, and 2.75-fold, respectively. *CYP6CW1* showed the highest expression level in the Donggang population (3.27-fold), followed by the Jiyang and Yutai populations (2.97- and 2.55-fold, respectively). *CYP425A1v2* also showed the highest expression level in the Donggang population (3.67-fold), followed by the Jiyang and Daiyue populations (2.91- and 2.88-fold, respectively). *CYP6CS2v1* was significantly over-expressed only in the Tancheng and Jiaxiang populations (3.82- and 2.83-fold). *CYP4CE2* only showed a remarkably high expression level in the Jiyang population (3.11-fold).

**Figure 3:**
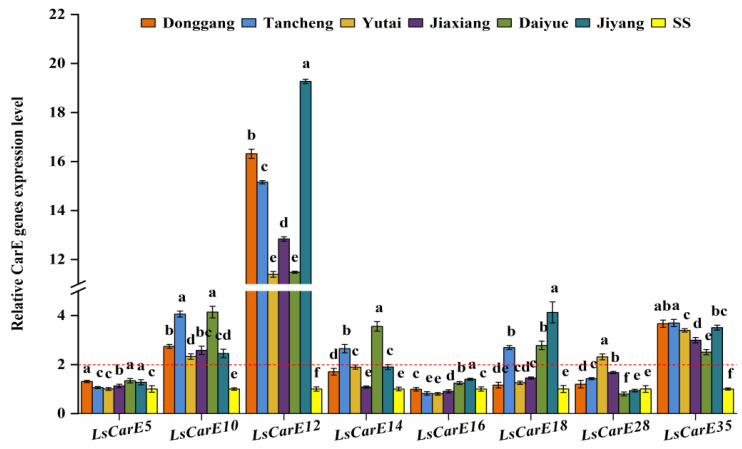
The relative expression levels of eight CarE genes in six *L. striatellus* populations from Shandong Province compared with SS (red dotted line means 2-fold overexpression level). The data are the mean ± SE (*n* = 3), and different letters on the bars indicate significant differences (*p* < 0.05).

As shown in Figure 3, compared to SS, the three CarE genes (*LsCarE10*, *LsCarE12*, and *LsCarE35*) were relatively over-expressed up to more than 2-fold in all six field populations, among which *LsCarE12* showed a high expression level of more than 10-fold change (from 11.39- to 19.27-fold). However, two CarE genes (*LsCarE5* and *LsCarE16*) were less than 2-fold in all the field populations. *LsCarE18* was significantly up-regulated in the Jiyang, Daiyue, and Tancheng populations with 4.13-, 2.78-, and 2.69-fold, respectively. *LsCarE14* also was significantly up-regulated in the Daiyue and Tancheng populations (3.56- and 2.65-fold, respectively). Only *LsCarE28* was significantly up-regulated in the Yutai population (2.31-fold).

**Figure 4:**
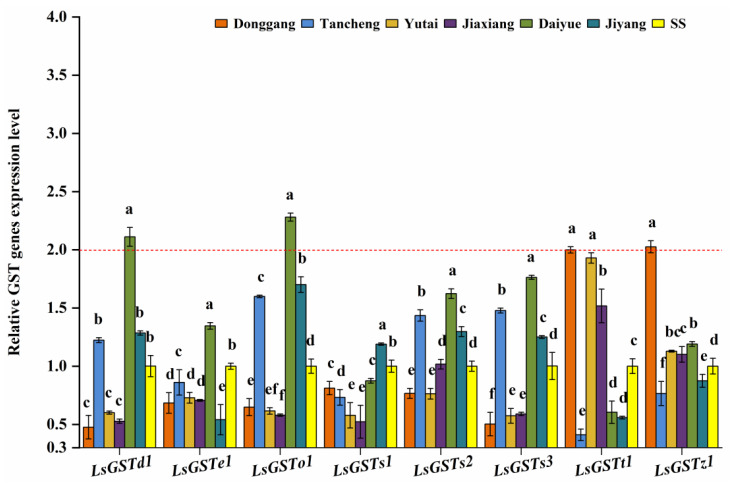
The relative expression levels of eight GST genes in six *L. striatellus* populations from Shandong Province compared with SS (red dotted line means 2-fold overexpression level). The data are the mean ± SE (*n* = 3), and different letters on the bars indicate significant differences (*p* < 0.05).

The relative expression levels of eight GST genes in all the field populations did not change much (Figure 4). Compared with SS, only two GST genes (*LsGSTd1* and *LsGSTo1*) in the Daiyue population, and other two GST genes (*LsGSTt1* and *LsGSTz1*) in the Donggang population were over-expressed up to more than 2-fold.

### 3.4. Gene Site Mutation in nAChR β1 Subunit in Different Populations of L. striatellus

According to the reported mutation sites that affect the sensitivity of *A. gossypii* to neonicotinoid insecticides, three sites V62I, R81T, and K264E of the nAChR β1 subunit in *L. striatellus* were tested for mutations. After comparing the partial amino acid sequences of the two species, it was found that the 62nd valine (V) and the 81st arginine (R) are in the same position of the nAChR β1 subunit sequence in the two species. However, the 264th lysine (K) site in *A. gossypii* should correspond to the 265th lysine (K) site of *L. striatellus*, because the latter has one more amino acid at the 218th position (aspartic) than the former.

The results of gene mutation detection showed that none of the three mutation sites (V62I, R81T, and K265E) were reported in the nAChR β1 subunit in any of the six field populations of *L. striatellus* in Shandong Province, and there were no mutations in SS either (Table 10).

## 4. Discussion

Currently, *L. striatellus* are mainly controlled by applying insecticides acting on nAChR, and had developed resistance to many insecticides, leading to control problems in the field [7,30]. The sensitivity determination results showed that three field populations exhibited multi-resistance to three tested insecticides (imidacloprid, dinotefuran, thiamethoxam), among which most showed low-level resistance, but the Donggang population had a medium-level resistance to imidacloprid. Low level resistance to sulfoxaflor was found only in the Tancheng population. However, all the field populations still showed sensitivity to nitenpyram, clothianidin, and triflumezopyrim, and the Daiyue and Jiaxiang populations did not develop resistance to all the tested insecticides. The insecticide multi-resistance of *L. striatellus* in the field had also been reported in many previous studies, which may be due to the history of pesticide application, and particular application habits and application levels in various regions [6,31,32]. In terms of geographical distribution, the four resistant populations (the Donggang, Tancheng, Jiaxiang, and Yutai populations) were all located in areas with abundant water resources, the climate was warm and humid, and the occurrence of *L. striatellus* was more serious [33]. Persistent excessive chemical control may be another important reason for insecticide resistance [34]. Therefore, a combination of imidacloprid with dinotefuran, sulfoxaflor, and thiamethoxam used in the field to control *L. striatellus* should be cautiously applied.

P450s, CarE, and GST are the three major metabolic enzymes in insects. Previous studies have confirmed that the enhanced activity of these three metabolic enzymes mediates insect resistance to insecticides acting on nAChR [5,35,36]. In this study, the contents of P450s, CarE, and GST in the Donggang, Yutai, and Daiyue were significantly higher than those in the Tancheng and Jiaxiang populations, and the enzyme contents were related to the sensitivity of the populations to imidacloprid, thiamethoxam, and dinotefuran. However, the enzyme activity cannot be directly inferred from enzyme contents, and the activities of these three enzymes still need to be detected in the future.

The overexpression of multiple P450 genes can jointly regulate the sensitivity of insects to insecticides [37,38]. Many previous studies have confirmed that *CYP314A1* contributes to the development of insecticide resistance in *N. lugens* and other insects [39,40]. Li et al. also found that the *CYP314A1* gene could affect the development, and increased the mortality of *L. striatellus* [41]. In this study, *CYP314A1* had the highest overexpression level in all tested populations of *L. striatellus*, but there were no significant correlations between the *CYP314A1* gene and insecticide sensitivity.

*CYP4C72* and *CYP6CW1* are associated with imidacloprid resistance in *L. striatellus* [42,43]. Similarly, our results showed that these two P450 genes were significantly up-regulated in some field populations. The above P450 genes may play an important role in insecticides resistance in *L. striatellus* populations in Shandong Province. In addition, *CYP6CS2v1*, *CYP425A1v2*, and *CYP4CE2* were significantly up-regulated at more than two-fold in some populations. However, a previous study found that the expression levels of these three genes were not different in the deltamethrin-resistant populations of *L. striatellus* [44].

Previous studies have found that *CarE12* and *CarE28* were significantly induced in *N. lugens* after treatment with sublethal concentrations of chlorpyrifos [45]. *CarE10* and *CarE18* were involved in the detoxification of fenfluthrin in *Tetranychus cinnabarinus* [46]. In this study, *LsCarE10* and *LsCarE12* were over-expressed in all tested field populations of *L. striatellus*, among which *LsCarE12* had the highest overexpression level. *LsCarE18* and *LsCarE28* were also up-regulated in some populations. A previous study showed that *CarE14* was significantly up-regulated in *T. cinnabarinus* after exposure to cyflumetofen [46]. The overexpression of *CarE14* was also observed in two field populations in our study. A previous study proved that *CarE5* and *CarE16* were induced to be up-regulated in *N. lugens* after treatment with nitenpyram [45], but the expression levels of these two genes were less than 2-fold in all the field populations in this study. We also found that *LsCarE35* was significantly up-regulated in all tested field populations, but there are no reports on whether this gene is related to insecticide resistance, and we will focus on this gene in the next study.

In this study, only *LsGSTd1* and *LsGSTo1* in the Daiyue population, and *LsGSTt1* and *LsGSTz1* in the Donggang population were significantly up-regulated. Previous studies have confirmed that *NIGSTd1* and *NIGSTz1* are overexpressed in the fipronil-resistant population of *N. lugens* [47]. Imidacloprid or chlorpyrifos resistance in *L. striatellus* associated with the up-regulated expression of *LsGSTo1* and *LsGSTt1* has also been reported [30,48]. However, changes in the expression levels of eight tested GST genes in all the populations were not obvious.

As a target of insecticides, mutations in the nAChR gene inactivate the activity of ion channels, resulting in reduced insect sensitivity to insecticides [49]. In this study, no reported mutations at the positions V62I, R81T, and K265E of the nAChR β1 subunit were found in the *L. striatellus* population in Shandong Province. This may be due to the low insecticide resistance level in the tested populations of *L. striatellus*. The reported mutations are often found in insects with high resistance level to insecticides targeted at nAChR [50,51].

## 5. Conclusions

This study shows that some *L. striatellus* populations in Shandong Province have developed resistance to imidacloprid, dinotefuran, sulfoxaflor, and thiamethoxam, and all the field populations are sensitive to clothianidin, nitenpyram, and triflumezopyrim. This differential sensitivity phenomenon is not currently associated with mutations at positions V62I, R81T, and K265E of the nAChR β1 subunit, and they may be related to the enhanced P450s, CarE, and GST contents and the overexpression of the related genes in *L. striatellus*.

## Figures and Tables

**Table 1:** Primers used for RT-qPCR and the nAChR β1 subunit amplification.

Gene	Accession Number	Forward Primer (5′-3′)	Reverse Primer (5′-3′)
*CYP4C72*	MG566048.1	GAAGACATTCGTGAGGAGG	CCCAAAAGTAGTAAGAGCCAG
*CYP4CE2*	MG566046.1	GCTCTTTCACCTTTCACCCAC	TTCATCACGTTCCAACTCCTC
*CYP6CS2v1*	JX876492.1	GAACAATCGCATCCAACAA	GGCATCCAAGTACTCCAGA
*CYP6CW1*	JX462960.1	ACTTCCAGTTTCAGACGCC	GACCGCTTCCCATCAGATC
*CYP314A1*	KC579461.1	CAAGGAGCGTCACAGAGTA	TCCATCACCAGAAGGAATAG
*CYP314A1v2*	JX566821.1	CCTATTCCTTCTGGTAATG	CGGCTTCTCTTCTATTATC
*CYP425A1v2*	JX876513.1	CCTACCAGAAAATATAAGCAAACGG	CACATTCCACATCAAACCTTCTG
*LsCarE5*	HM600727.1	CTTGTGGCAGGATTCGTAGAG	TCATGATTATCACCGAGAAGCC
*LsCarE10*	JQ990753.1	CAAAATGAGCAGGGAATCGC	TTGGATTCAGAGGTGTGGC
*LsCarE12*	JQ990755.1	CCGCTTCCTGTCATTTTCT	TGTTCTCAACGTAGGCCCA
*LsCarE14*	JQ990757.1	TCTGAGGACTGTCTCTATCTGG	CCACGTCAGCCTCTTTACTATC
*LsCarE16*	MK238305.1	GGTGCGGAATTCGTTGAAAAC	GGTGACTCGTTAATTGGTTTGC
*LsCarE18*	JQ990761.1	AAGCAAGCTGAGCAGATCG	CTTCAACAAGTCGTAGGGA
*LsCarE28*	JQ990771.1	GCCTGACAAAATGCTCTCAAG	GGCAATGTTCTGTTTCACCC
*LsCarE35*	JX566828.1	CTCTATGGGTTATGTCTTCGCG	CTCAACTCTACAATCGGAGGC
*LsGSTd1*	JN628446.1	TTCCCAGTTGTAAGGCTTGG	CAAGAGTCGATATAGATGCGGC
*LsGSTe1*	JN628441.1	GCCGGTGATCAGATGACTATC	TGGCCGAATCGTAATCCTTC
*LsGSTo1*	JN628448.1	TCCCAAGTGCTTTATACTCATAGG	CTAAAGGTGGGTCTGTGGAG
*LsGSTs1*	JN628440.1	TTCAATGCTAGAGGAAGAGCG	TGCCATCCACTTCTAAAACAGG
*LsGSTs2*	JN628443.1	GCATCAAAATTCAGCTCAGTCG	TCCAGATAAGACAGCAACCATC
*LsGSTs3*	JN628444.1	GCAATCAGTCGCCATTTCTAG	GCTTCATCGCTTTCATAGAACC
*LsGSTt1*	JN628445.1	CCACCCAATCAAGAAACAGTTAAT	ATCATAACCGGCCATTCTGG
*LsGSTz1*	JN628442.1	GGCTAAGGTGAGGGAGATTTG	CCGACACAATATTTGCCAGC
GAPDH	HQ385974.1	GTGTTGACTACATGGTCTACT	GCTCACTGAATACCTGGATT
nAChR β1	MF612140.1	GTTCTGCTAGTCTTCGGAGTC	TTCCCGAATCTGTATGTACTG

**Table 2:** LC_50_ values of the susceptibility baseline of *L. striatellus*.

Insecticide	LC_50_ (95% CI) (mg/L)	Reference
Imidacloprid	9.3060 (7.1210–11.5550)	[28]
Nitenpyram	1.2310 (0.9350–1.5310)	[29]
Thiamethoxam	1.7920 (1.3390–2.2770)	[29]
Dinotefuran	0.5280 (0.3690–0.6980)	[28]
Sulfoxaflor	0.3980 (0.2880–0.5030)	[21]
Clothianidin	2.5000 (1.6918–3.6945)	Present study
Triflumezopyrim	0.4867 (0.3525–0.6672)	Present study

**Table 3:** Sensitivity to imidacloprid in six *L. striatellus* populations in Shandong Province.

Population	LC_50_ (mg/L)	95% CI (mg/L)	Slope ± SE	*χ*^2^ (df)	R^2^	RR
Donggang	162.67	87.40–264.68	1.28 ± 0.32	2.26 (3)	0.89	17.48
Tancheng	29.49	21.03–46.97	1.57 ± 0.30	4.00 (3)	0.88	3.17
Yutai	67.26	46.95–115.03	1.41 ± 0.27	3.03 (3)	0.92	7.23
Jiaxiang	23.63	16.26–36.61	1.48 ± 0.30	1.77 (3)	0.94	2.54
Daiyue	42.14	24.19–71.27	1.14 ± 0.35	2.36 (3)	0.82	4.53
Jiyang	31.99	23.42–46.77	1.72 ± 0.31	1.98 (3)	0.94	3.44

**Table 4:** Sensitivity to dinotefuran in six *L. striatellus* populations in Shandong Province.

Population	LC_50_ (mg/L)	95% CI (mg/L)	Slope ± SE	*χ*^2^ (df)	R^2^	RR
Donggang	3.08	2.14–4.77	1.45 ± 0.29	4.34 (3)	0.86	5.84
Tancheng	2.24	1.44–3.35	1.39 ± 0.29	3.28 (3)	0.89	4.25
Yutai	4.12	3.18–6.21	2.66 ± 0.49	4.50 (3)	0.89	7.81
Jiaxiang	1.93	1.35–2.58	1.84 ± 0.31	7.95 (3)	0.84	3.65
Daiyue	2.57	1.67–4.00	1.68 ± 0.37	2.40 (3)	0.89	4.86
Jiyang	2.66	2.10–3.42	2.55 ± 0.38	7.30 (3)	0.90	5.04

**Table 5:** Sensitivity to sulfoxaflor in six *L. striatellus* populations in Shandong Province.

Population	LC_50_ (mg/L)	95% CI (mg/L)	Slope ± SE	*χ*^2^ (df)	R^2^	RR
Donggang	1.55	1.13–2.39	1.63 ± 0.28	1.29 (3)	0.96	3.90
Tancheng	2.07	1.42–2.85	1.57 ± 0.28	3.30 (3)	0.92	5.20
Yutai	1.62	1.12–2.13	1.95 ± 0.31	8.43 (3)	0.84	4.07
Jiaxiang	1.41	1.04–1.99	1.87 ± 0.33	6.84 (3)	0.85	3.54
Daiyue	1.79	1.18–2.48	1.54 ± 0.28	3.07 (3)	0.92	4.50
Jiyang	2.13	1.41–3.04	1.41 ± 0.27	0.81 (3)	0.98	5.35

**Table 6:** Sensitivity to thiamethoxam in six *L. striatellus* populations in Shandong Province.

Population	LC_50_ (mg/L)	95% CI (mg/L)	Slope ± SE	*χ*^2^ (df)	R^2^	RR
Donggang	6.87	5.15–9.92	1.92 ± 0.33	5.84 (3)	0.87	3.83
Tancheng	6.37	4.32–8.44	2.10 ± 0.35	3.99 (3)	0.80	3.55
Yutai	12.58	8.75–19.73	1.52 ± 0.31	1.21 (3)	0.97	7.02
Jiaxiang	5.12	3.68–7.16	1.90 ± 0.35	7.52 (3)	0.81	2.86
Daiyue	7.77	5.63–12.24	1.70 ± 0.31	6.14 (3)	0.87	4.34
Jiyang	6.57	4.80–9.77	1.81 ± 0.34	3.56 (3)	0.90	3.66

**Table 7:** Sensitivity to nitenpyram in six *L. striatellus* populations in Shandong Province.

Population	LC_50_ (mg/L)	95% CI (mg/L)	Slope ± SE	*χ*^2^ (df)	R^2^	RR
Donggang	1.33	0.98–1.93	1.67 ± 0.28	1.55 (3)	0.96	1.08
Tancheng	0.68	0.51–0.98	1.76 ± 0.29	1.52 (3)	0.97	0.55
Yutai	2.01	1.37–3.80	1.39 ± 0.28	5.09 (3)	0.86	1.63
Jiaxiang	0.78	0.51–1.49	1.15 ± 0.26	4.96 (3)	0.80	0.63
Daiyue	1.08	0.81–1.48	1.78 ± 0.29	0.47 (3)	0.99	0.88
Jiyang	0.95	0.66–1.70	1.43 ± 0.28	5.32 (3)	0.85	0.77

**Table 8:** Sensitivity to clothianidin in six *L. striatellus* populations in Shandong Province.

Population	LC_50_ (mg/L)	95% CI (mg/L)	Slope ± SE	*χ*^2^ (df)	R^2^	RR
Donggang	3.97	2.67–5.54	1.51 ± 0.27	3.75 (3)	0.86	1.59
Tancheng	3.52	2.59–5.27	1.76 ± 0.31	2.76 (3)	0.89	1.41
Yutai	3.76	2.66–5.00	1.81 ± 0.29	1.42 (3)	0.89	1.51
Jiaxiang	3.50	2.46–5.62	1.48 ± 0.29	3.44 (3)	0.84	1.40
Daiyue	5.61	3.96–8.26	1.87 ± 0.36	2.09 (3)	0.89	2.24
Jiyang	3.56	2.16–5.17	1.32 ± 0.27	1.74 (3)	0.90	1.42

**Table 9:** Sensitivity to triflumezopyrim in six *L. striatellus* populations in Shandong Province.

Population	LC_50_ (mg/L)	95% CI (mg/L)	Slope ± SE	*χ*^2^ (df)	R^2^	RR
Donggang	0.99	0.69–1.42	1.47 ± 0.27	5.99 (3)	0.85	2.03
Tancheng	0.69	0.47–0.93	1.8193 ± 0.31	6.97 (3)	0.87	1.42
Yutai	0.89	0.63–1.20	1.90 ± 0.33	6.69 (3)	0.86	1.82
Jiaxiang	0.61	0.45–0.88	1.63 ± 0.28	2.44 (3)	0.94	1.26
Daiyue	0.76	0.50–1.08	1.52 ± 0.29	1.94 (3)	0.94	1.57
Jiyang	0.67	0.47–0.89	1.97 ± 0.32	3.17 (3)	0.94	1.38

**Table 10:** The nAChR β1 subunit gene mutation sites and frequency in six *L. striatellus* populations from Shandong Province.

Population	Insects	Frequency of Mutation Site		
		V62I	R81T	K265E
Donggang	30	0	0	0
Tancheng	30	0	0	0
Yutai	30	0	0	0
Jiaxiang	30	0	0	0
Daiyue	30	0	0	0
Jiyang	30	0	0	0
SS	30	0	0	0

## Data Availability

The data supporting the conclusion of this paper have been reflected in the main text. The original data can be obtained from the corresponding authors.

## Supplementary Materials

The following supporting information can be downloaded at: https://www.mdpi.com/article/10.3390/insects13090780/s1, S1: The serial concentrations of each pesticide tested; S2: Determination steps of metabolic enzymes contents.
