# Supplementary material for: Sensitivity Differences and Biochemical Characteristics of Laodelphax striatellus (Fallén) to Seven Insecticides in Different Areas of Shandong, China
Source: Insects. 2022 Aug 29;13(9):780. doi: 10.3390/insects13090780 (PMC9506532; doi:10.3390/insects13090780)
Supplement: Supplementary file 1 [file insects-13-00780-s001.zip › insects-1834291-supplementary.pdf]

Supplementary Materials

S1. The serial concentrations of each pesticide tested

The serial concentrations were set as follows: imidacloprid: 50, 100, 200, 400, 800 mg/L (Donggang population), 10, 20, 40, 80, 160 mg/L (the other five tasted populations); thiamethoxam: 2.5, 5, 10, 20, 40 mg/L (six tested populations); dinotefuran: 0.625, 1.25, 2.5, 5, 10 mg/L (six tested populations); sulfoxaflor: 0.5, 1, 2, 4, 8 mg/L (six tested populations); nitenpyram and triflumezopyrim: 0.25, 0.5, 1, 2, 4 mg/L (six tested populations); clothianidin: 1, 2, 4, 8, 16 mg/L (six tested populations).

S2. Determination steps of metabolic enzymes contents

The P450s, CarE, and GST contents were detected according to the kit's instructions (Hengyuan Biotechnology Co., Ltd., Shanghai, China). Thirty third-instar nymphs were homogenized with 300 μL of 0.01 M phosphate buffer (pH 7.6) in a 1.5 mL centrifuge tube. After centrifugation at 4 °C and 10000×g for 10 min, the supernatant was pipetted into a new centrifuge tube as the enzyme solution to be tested. Three replicates were set for each population (30 insects per replicate).

The determination steps of P450s contents were as follows: First, the 50 μL of 5-fold diluted enzyme solution was added to the sample wells of a 96-well microtiter plate coated with the special P450s antibody, the P450s standard solution was diluted to 8.1875, 16.375, 32.75, 65.5, 131 μg/g with sample diluent and added to standard wells of the plate. No samples and reagents were added to blank control wells. Then, the plate was covered with sealing film and was incubated at 37 °C for 25 min for specific binding of antibody-antigen. After incubation, the solution was discarded and the plate was washed 5 times with 30-fold diluted washing solution. After being dried, 50 μL horseradish peroxidase (HRP) -conjugated P450s antibody reagent was added to the above wells and incubated at 37 °C for 25 min for specific binding of antibody–antigen–antibody. After being washed and dried, the 50 μL of chromogen solution A (buffer solution) and solution B (tetramethylbenzidine, TMB) was added in turn and mixed, then incubated at 37 °C in the dark for 15 min to generate color. Finally, the 50 μL of stop solution (1 M sulfuric acid) was added to terminate the reaction. The OD values at 450 nm were measured using a microplate reader (BioTek Instruments, Inc, Highland Park, USA).

The CarE and GST contents were also determined by the assay kits following the same steps of P450s detection. However, the 96-well microtiter plates used for CarE and GST were coated with the special CarE antibody and GST antibody, respectively. In addition, following the kit's instructions, the CarE standard solution was diluted to 8.1875, 16.375, 32.75, 65.5, 131 μg/g, and the GST standard solution was diluted to 4.875, 9.75, 19.5, 39, 78 μg/g. The protein standard solution was diluted to serial concentrations and mixed with the enzyme solution, and then BCA working solution was added. After incubation at 37 °C for 25 min, the OD values at 562 nm were measured using an Epoch 2 multi-function microplate reader (BioTek Instruments, Inc, Highland Park, USA).

The enzyme contents were calculated according to the standard curve (P450: y=0.0531x-0.0297; CarE: y=0.0463x-0.006; GST: y=0.0556x-0.0162) along with the protein contents (Donggang population: 1.68 g/L; Tancheng population: 1.64 g/L; Yutai population: 1.89 g/L; Jiaxiang population: 1.63 g/L; Daiyue population: 1.93 g/L; Jiyang population: 1.63 g/L); the results are expressed as μg/g prot.
